# Macroporous Calcium Phosphate/Chitosan Composites Prepared via Unidirectional Ice Segregation and Subsequent Freeze-Drying

**DOI:** 10.3390/ma10050516

**Published:** 2017-05-08

**Authors:** Inmaculada Aranaz, Enrique Martínez-Campos, Carolina Moreno-Vicente, Ana Civantos, Sara García-Arguelles, Francisco del Monte

**Affiliations:** 1 Instituto de Ciencia de Materiales de Madrid-ICMM, Consejo Superior de Investigaciones Científicas-CSIC, Cantoblanco 28049, Madrid, Spain; sara_g.arguelles@yahoo.es; 2 Tissue Engineering Group, Institute of Biofunctional Studies, Associated Unit to the Institute of Polymer Science and Technology (CSIC), Pharmacy Faculty, Complutense University of Madrid (UCM), Paseo Juan 23, n1 28040, Madrid, Spain; emartinezcampos79@gmail.com (E.M.-C.); carolmv@gmail.com (C.M.-V.); anacivantos@gmail.com (A.C.); 3 Departamento de Tecnología Química y Energética, Tecnología Química y Ambiental y Tecnología Mecánica y Química Analítica, Universidad Rey Juan Carlos, Móstoles 28933, Madrid, Spain

**Keywords:** biomineralization, amorphous calcium phosphate, hydroxyapatite, chitosan, biocompatibility

## Abstract

Calcium phosphate chitosan-based composites have gained much interest in recent years for biomedical purposes. In this paper, three-dimensional calcium phosphate chitosan-based composites with different mineral contents were produced using a green method called ice segregation induced self-assembly (ISISA). In this methodology, ice crystals were used as a template to produce porous structures from an aqueous solution of chitosan (CS) and hydroxyapatite (Hap) also containing acetic acid (pH = 4.5). For better characterization of the nature of the inorganic matter entrapped within the resulting composite, we performed either oxygen plasma or calcination processes to remove the organic matter. The nature of the phosphate salts was studied by XRD and NMR studies. Amorphous calcium phosphate (ACP) was identified as the mineral phase in the composites submitted to oxygen plasma, whereas crystalline Hap was obtained after calcination. SEM microscopy revealed the formation of porous structures (porosity around 80–85%) in the original composites, as well as in the inorganic matrices obtained after calcination, with porous channels of up to 50 µm in diameter in the former case and of up to 20 µm in the latter. The biocompatibility of the composites was assessed using two different cell lines: C2C12GFP premyoblastic cells and MC3T3 preosteoblastic cells.

## 1. Introduction

Hydroxyapatite (Hap) is a naturally occurring calcium phosphate, the chemical structure of which is (Ca_5_(PO_4_)_3_OH). Among other calcium phosphates, Hap is the most thermodynamically stable and presents interesting properties such as excellent biocompatibility, and favorable osteoconductive and bioactive properties. It is therefore widely preferred as the biomaterial of choice in dentistry, orthopaedics and regenerative medicine [1,2]. Chemically, Hap bioceramics closely resemble the inorganic component of natural bone [3]. Calcium phosphate materials included in natural bone have some specific characteristics such as mineral nanophase, poorly crystallized nature and the presence of non-stoichiometric apatite phase. This mineral phase contains different ions such as carbonate, sodium, and fluoride, among others, in a polymeric fibre matrix of collagen. Hap composites with polymers and metals have been developed to improve the mechanical properties of porous Hap or to produce partially biodegradable artificial bone grafts for tissue engineering [4,5]. Moreover, surface topography favors cell adhesion, and Ca^2+^ and (PO_4_)^3−^ release from these scaffolds regulates bone cell migration, proliferation and differentiation [6]. With regard to the role played by Ca^2+^ and (PO_4_)^3−^ release in bone regeneration, i.e., based on the dissolution of calcium phosphate precursor and the subsequent recrystallization into bone tissue, nanocomposites based on nanoparticles of amorphous calcium phosphate (ACP) would be of even greater interest than those based on nanocrystalline Hap [7,8,9].

Chitosan (CS) is a linear polymer of natural origin composed of randomly distributed β-(1→4)-linked d-glucosamine (deacetylated unit) and N-acetyl-d-glucosamine (acetylated unit). CS is a cationic polymer, soluble in acid aqueous media but not at physiological pH. It is biodegradable, biocompatible and non-toxic, and therefore it has been used in a wide range of biomedical applications, such as bone and cartilage tissue engineering, due to its structural similarity to GAGs (glycosaminoglycan’s) [10,11]. From a technological point of view, CS is very versatile and with it materials with different configurations (porous scaffolds, fibres and so on) can be easily produced [12]. Moreover, CS has been incorporated into Ca-P coatings using electrochemical deposition [13]. In fact, CS-Hap composites have gained interest, since it is possible to combine the properties of both materials avoiding the disadvantages of Hap, such as its brittleness. These composite materials have demonstrated good biocompatibility and osteocompatibility.

**Scheme 1:**
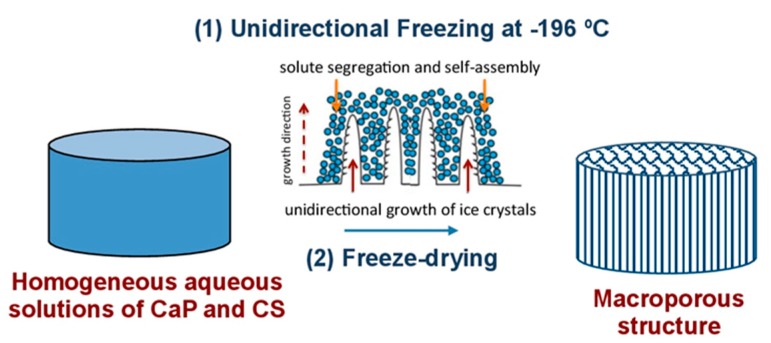
Macroporous three-dimensional structures produced via unidirectional ice segregation of a solution of chitosan containing dissolved Hap, and subsequent freeze-drying. Adapted from References [14,15].

Most of the processes designed to produce foams and scaffolds make use of solvents and templates that need to be removed prior to the material use in applications such as biomedicine. Removal of these chemical compounds is not a trivial issue, and cryogenic processes that make use of ice as a friendly template provides an interesting alternative to conventional chemical solvents and templates [14]. Our group coined the term ISISA (ice segregation induced self-assembly) to refer to the preparation of inorganic, organic, and hybrid materials with well-patterned macroporous structures via the unidirectional freezing of aqueous suspensions and hydrogels in liquid nitrogen [15]. This ice formation causes most solutes originally dispersed in the aqueous suspension to be segregated from the ice phase, giving rise to a macroporous structure characterised by “fences” of matter enclosing ice. The scaffolds obtained after subsequent drying (by both simple thawing and freeze-drying) show a macroporosity that corresponds to the empty areas where ice crystals originally resided (Scheme 1).

In this paper, macroporous three-dimensional structures were produced via ISISA of a solution of chitosan containing dissolved Hap, and these structures were subsequently freeze-dried with the aim of developing a material with properties closer to those of living bone, such as mineral nanophase and monolithic structure. 

## 2. Results

As mentioned in the introduction, we produced macroporous three-dimensional structures composed of calcium phosphate homogeneously dispersed in a chitosan matrix using a cryogenic process known as ice segregation induced self-assembly (ISISA). The experimental conditions used for the preparation of a set of CSCaP composites are shown in Table 1. Three monoliths (denoted as samples CSCaP1, CSCaP2, and CSCaP3) with different CaP contents were produced. The mineral content of the monoliths was confirmed by thermogravimetric studies (Figure S1). Moreover, Thermogavimetric analysis (TGA) scans revealed how weight loss followed a common trend for every sample. The first weight loss occurred around 50–70 °C and was assigned to humidity loss (Table 1). The second one happened around 250–270 °C and was assigned to chitosan deacetylation and degradation. Finally, the third one appeared around 440–470 °C and was assigned to the oxidative decomposition of the carbonaceous residue produced in the second thermal event [16]. It is worth noting that Hap has two types of water in its structure—adsorbed and lattice water. Adsorbed water is irreversibly lost in a temperature of 25–200 °C without any effect on lattice parameters. On the contrary, lattice water is irreversibly lost at a temperature of 200–400 °C and causes a contraction in the a-lattice dimension during heating. Typically, no further weight loss has been described in the temperature range of 400–800 °C [17]. The agreement of CSCaP1 and CSCaP2 between residual weight and mineral content included during monolith preparation revealed that the abovementioned weight loss mostly corresponded to chitosan decomposition. Meanwhile, weight loss in CSCaP3 extended up to 525 °C, most likely due to some delayed CaP dehydration. Actually, mineral content was around 25% at 470 °C, and this amount is close to that which corresponds to the amount of Hap originally included during monolith preparation.

**Figure 1:**
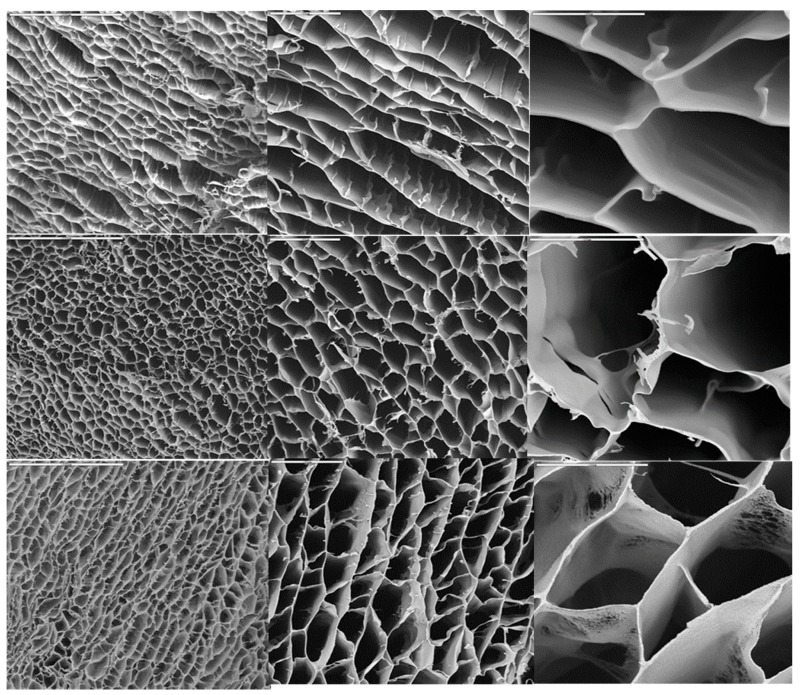
SEM micrographs of different hierarchical structures resulting from freezing samples with different CS/CaP ratios. CSCaP1 (**upper line**), CSCaP2 (**middle line**) and CSCaP3 (**bottom line**). From left to right, bars are 200, 50 and 20 µm, respectively.

The structure of the macroporous hierarchical structures is shown in Figure 1. In all cases, pore diameters were around 20–50 µm. The sample with the lowest CaP content (ca. 6.25%) exhibited a lamellar-like morphology with low interconnectivity among layers. As the concentration was increased, the structure became honeycomb-like as consequence of the enhanced interconnectivity among layers. The porosity was around 80–85% for every sample no matter the initial Hap content. In particular, the porosity for CSCaP1, CSCaP2 and CSCaP3 was 83.4, 80.1 and 84.6, respectively. 

**Figure 2:**
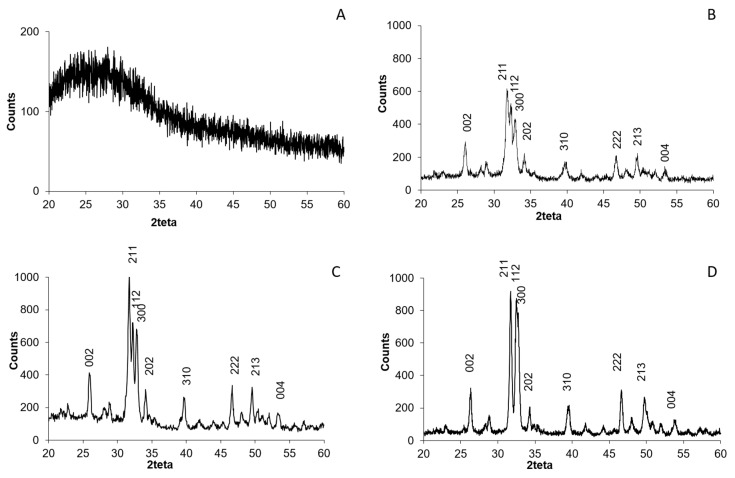
XRD pattern of CSCaP3 (**A**); CSCaP1C (**B**); CSCaP2C (**C**) and CSCaP3C (**D**) samples.

CSCaP composites were also studied by XRD (Figure 2A). Unfortunately, the composites exhibited no crystalline features, i.e., the broad band centered at ca. 25 degrees corresponded to the sample holder (silica substrate) used for XRD. The lack of chitosan crystallinity is due to the presence of acetic acid, which may hinder the formation of inter- and intramolecular hydrogen bonds in chitosan and avoid packaging in an ordered fashion. Meanwhile, any crystalline feature coming from CaP was undetectable even in the sample with the highest mineral content, CSCaP3. For comparison, we performed the XRD of a physical mixture of CS and Hap in the wt % used for CSCaP3 preparation (Figure S2). In this case, peaks corresponding to crystalline Hap (Hap standard ICDD-PDF-00-024-0022) were perfectly visible in the diffraction pattern. Based on this, the lack of crystalline features in CSCaP1, CSCaP2 and CSCaP3 revealed the amorphous nature of CaP salts or, alternatively, the minor presence of very small Hap crystals. Attempts aiming to obtain further insights about the CaP features in CSCaP samples—e.g., by TEM, FTIR, etc.—failed because the major content of chitosan masked any potential CaP response. 

Thus, we performed two different treatments—e.g., oxygen plasma (samples denoted as CSCaPOP) and calcination (samples denoted as CSCaPC)—to eliminate the polymer, either partially or totally, and thus favor the study of the mineral residue. The former consisted of the room-temperature exposure of the composites to oxygen plasma. This process is typically used for the removal of organic impurities from two-dimensional surfaces. In biomaterials, oxygen plasma has also been used for either sterilization purposes or functionalization of the organic component of the biomaterial [18,19]. In all these works, the mineral phase remained unmodified after oxygen plasma treatment. In our case, the open macropore structure of our samples allowed the use of oxygen plasma for CS removal under experimental conditions—low pressure and temperatures below 100 °C—where the nature of the mineral phase remained basically unaltered. After oxygen plasma treatment, polymer removal ranged between 40% and 88% in the resulting samples, e.g., from CSCaP3OP to CSCaP1OP. The second approach followed for full CS removal was, according to previous TGA scans, calcination at 650 °C in air atmospheres so that the residue just corresponded to the mineral phase in the resulting samples, e.g., CSCaP1C, CSCaP2C and CSCaP3C.

The Fourier transform infrared spectrum (FTIR) of CSCaPOP samples (Figure S3) revealed the presence of calcium phosphate, e.g., bands at around 606 and 563 cm^−1^ (ν4). The assignment of further signals was difficult because of overlap with those coming from chitosan. The FTIR spectrum of CSCaPC samples also exhibited phosphate bands, e.g., at around 1088 and 1036 cm^−1^ (ν3), 960 (ν1) and 601 and 570 (ν4). A wide absorption band within the range from ~3600 cm^−1^ up to 3100 cm^−1^ points on ν3 and ν1 with H_2_O molecules bonded with hydrogen for stretching modes and an absorption band at 1629 cm^−1^ referable onto the deformation mode ν2 of H_2_O molecules proves the presence of physically adsorbed water in the synthesized samples [20]. The signal at 874 cm^−1^ could be ascribed to either HPO_4_^2−^, thus denoting the presence of non-stoichiometric Hap, or to carbonate. Signals observed at 1422 and 1468 cm^−1^ along with the signal at 874 cm^−1^ are typical of carbonate [21]. No signal from chitosan (around 1550 cm^−1^) was observed due to the amide I, which indicates that the polymer was effectively eliminated during calcination. This result is in good agreement with thermogravimetric analysis (Figure S1, Table 1). From FTIR analysis, it was possible to determine the splitting factor (SF) [22]. This parameter has been related to crystalline Hap and increases along with the crystallinity. As seen in Table 2, the SF increased from CSCaP1C to CSCaP2C in good agreement with the XRD analysis. In sample CSCaP3C, the SF value was similar to CSCaP1C.

The XRD pattern of CSCaPOP samples did not reveal any characteristic diffraction for crystalline Hap (Figure S2B). On the contrary, the XRD of CSCaPC samples revealed the typical XRD pattern of crystalline Hap (Hap standard ICDD-PDF-00-024-0022) with diffraction peaks at 2θ values of 25.9, 31.7, 32.9, 39.8, 46.7, 49.5, and 53.1, which are indexed to (002), (211), (300), (310), (222), (213) and (004) planes, respectively (Figure 2B–D) [23]. The degree of crystallinity (*Xc*) (Table 2) increased along with the original CaP content in the composites. It was not possible to determine *Xc* of the sample CSCaP3C (original CaP content was 30%) since the peaks used to determine *Xc* (valley between 112 and 300 planes, see Materials and Methods for more details) were not properly defined. The crystallite size measured from the XRD data was lower than 25 nm in the three samples.

**Figure 3:**
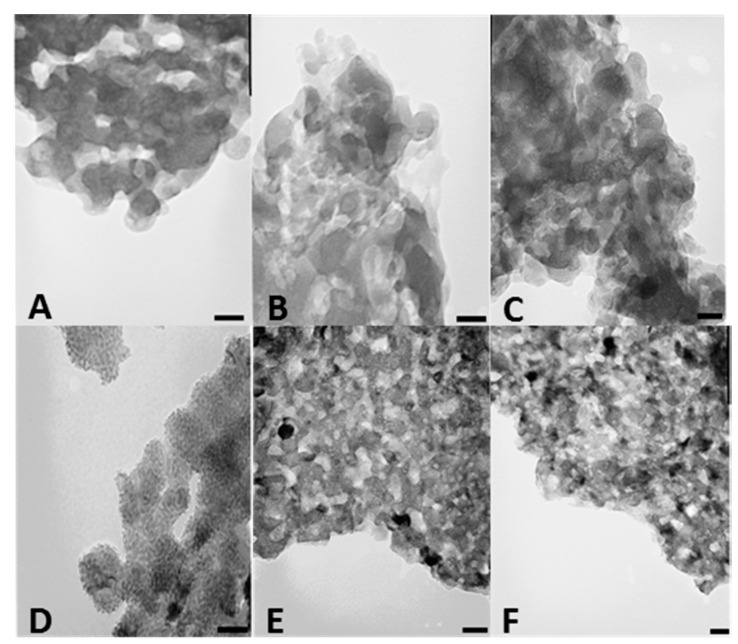
TEM micrographs of CSCaP1OP (**A**); CSCaP2OP (**B**); CSCaP3OP (**C**); CSCaP1C (**D**); CSCaP2C (**E**) and CSCaP3C (**F**). Bars are 50 nm.

We also investigated CSCaPOP and CSCaPC samples by transmission electron microscopy (TEM). TEM micrographs of CSCaPOP samples (Figure 3A–C) showed calcium phosphate particles aggregated in clusters of around 25–50 nm in diameter embedded in the chitosan matrix remaining after oxygen plasma treatment. CSCaPC samples exhibited a more crystalline-like morphology with a significant reduction in particle diameter as compared to CSCaPOP samples (see Table 2). Among the different CSCaPC samples, particles in CSCaP1C exhibited a lower dispersion in diameter than particles in both CSCaP2C and CSCaP3C, where the higher mineral content favored aggregation (Figure 3D–F).

**Figure 4:**
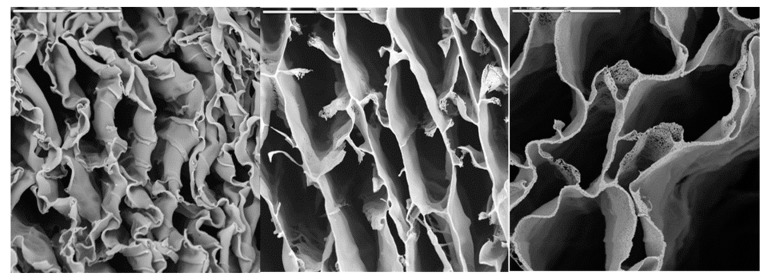
SEM micrographs of different hieratical structures resulting from the calcination of CSCaP nanocomposites. From left to right: CSCaP1C, CSCaP2C and CSCaP3C. Bars are 20 µm.

We finally investigated whether CSCaPOP and CSCaPC samples were capable of preserving a monolithic structure after their respective treatments. In this regard, CS removal was by no means trivial in CSCaOP samples, and the monolithic structure collapsed no matter the mineral content. This was not the case for CSCaPC samples where the formation of crystals and their subsequent aggregation allowed for the preservation of the structural integrity of the monoliths, obviously with certain shrinkage as compared to the original composites. In fact, this was observed by scanning electron microscopy (SEM), as the deformation caused by shrinkage decreased along with the mineral content of the original composite, i.e., more so for CSCaP1C than for CSCaP2C and CSCaP3C (Figure 4). 

The above study of CSCaPOP and CSCaPC samples revealed some interesting features. In agreement with previous works, CS removal in CSCaPOP provided negligible changes in the nature of the mineral phase [18]. For instance, XRD confirmed the non-crystalline features of the calcium phosphate salts. Meanwhile, calcination indeed promoted a significant change in the original nature of the CaP salts and crystalline Hap was observed in every sample—see FTIR and XRD.

**Figure 5:**
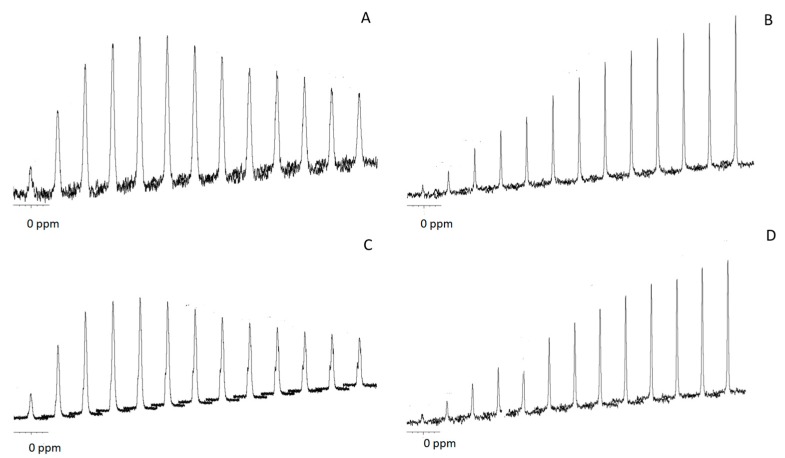
Array of CP spectra at different contact times for CSCaP1 (**A**); CSCaP1C (**B**); CSCaP3 (**C**) and CSCaP3C (**D**). From left to right contact times: 0.1, 0.5, 1, 1.5, 2, 3, 4, 5, 6, 7, 8, 9 and 10 ms.

For the purpose of elucidating further differences between the calcium phosphate salts in the original and calcined samples, we studied both samples by ^31^P NMR spectroscopy (Figure S4). The isotropic ^31^P chemical shift (ppm) measured by magic angle sample spinning NMR (MAS NMR) was around 1.5 and 3.1 in CSCaP and CSCaPC samples, respectively. We also performed ^1^H→^31^P cross-polarization (CP) for different contact times (Figure 5). CP allows for the enhancement of low-intensity solid-state NMR signals from dilute spins by polarization transfer from abundant spins. Since CP relies on heteronuclear dipolar couplings between the source and target spins, its efficiency is uniquely dependent on the concentration, distribution and mobility of the nuclei involved. In particular, CP has been widely used to obtain insights about the composition of different calcium phosphate salts and thus distinguish them—for instance, bone apatites from synthetic ones [24,25], or ACP salts from crystalline ones [26]—based on the different percentages of water molecules and hydroxyl groups that characterize each particular calcium phosphate type. In the particular case of crystalline apatites and amorphous calcium phosphate salts, CP efficiency is typically linear for contact times longer than 8 ms in the former case, whereas it reaches a maximum earlier in the latter because there are fewer apatite-structural-hydroxyl groups than water molecules occupying the interstices among the ACP clusters [26]. This was actually our case, with the CP efficiency growing linearly up to contact times of 10 ms for CSCaPC1 and CSCaPC3, and reaching a maximum for contact times of 1.5–2 ms for CSCaP1 and CSCaP3. It is worth noting that the TEM micrograph of CSCaPOP, in which CS removal provided negligible changes in the nature of the mineral phase, well resembled the characteristic cluster morphology of ACP salts.

**Figure 6:**
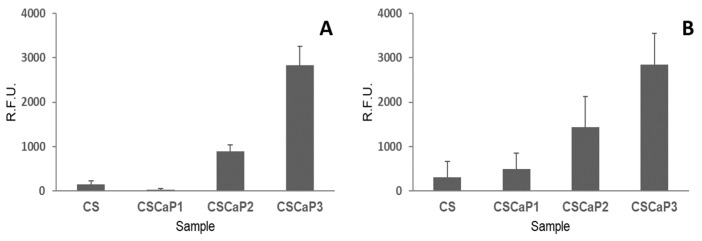
Metabolic activity (Alamar Blue) of cell cultures over CSCaP composites and control (CS monolith). (**A**) Premyoblastic C2C12GFP cell culture at 7 days and (**B**) osteoblastic MC3T3 cell culture at 7 days. R.F.U: Relative Fluorescent Units. *N* = 3.

Once the nature of the calcium phosphate included in the CS matrix was revealed, the next step was to determine the biocompatibility of CSCaP composites using two different cell lines: C2C12GFP premyoblastic cells (cells modified to include a fluorescent marker, see Materials and Methods section for more details) and MC3T3 preosteoblastic cells. At 7 days, the metabolic activity of the seeded cells on the supports (CSCaP composites and CS scaffold) was analyzed in both cell cultures (Figure 6). C2C12GFP and MC3T3 cell cultures showed a similar trend. First, low metabolic activity values for CS control and CSCaP1 composites were obtained for both cell lines. Although no specific signs of cell death were detected in these samples, only isolated cell clumps were observed in the surface and into the porous structures (Figures S5 and S6). The cell attachment of the premyoblastic C2C12GFP cell line was poor, with rounded morphologies slightly adhered to the surface. The MC3T3 osteoblastic cell line results were similar, with a low cell activity over these composites. An intermediate situation was found for CSCaP2, with small cell patches proliferating in these samples. In this case, premyoblastic and preosteoblastic cell monolayers were found growing on the surfaces and inside the inner structures. Finally, CSCaP3 showed good cytocompatibility behavior for both cell lines, with high metabolic activity readings. In this sample, higher cell densities and larger cell monolayers with an outstretched cell morphology were detected. 

## 3. Discussion

In this paper, we have described the preparation of macroporous CS scaffolds containing CaP salts. The samples were produced by mixing CS and Hap solutions followed by a unidirectional freeze-drying process (so-called ice segregation induced self-assembly, ISISA). With this methodology, macroporous scaffolds with an excellent control of the structural characteristics (Figure 1) and a homogeneous distribution of the mineral phase were easily prepared. TGA analysis was in good agreement with the CS/CaP mass ratio used for monoliths preparation (Table 1). After sample calcination at 650 °C, the macroporous structure was kept and pure CaP macroporous structures were produced (Figure 4).

FTIR spectra of samples exhibited the typical signals assigned to CaP (Figure S3). After calcination, the intensity of the signals ascribed to the polymer was negligible, confirming the lack of remaining polymer as anticipated by TGA analysis. The presence of carbonate in calcined samples pointed to the formation of non-stoichiometric Hap, and this is the natural phase found in bone. 

XRD patterns of CSCaP and CSCaPC samples were investigated to determine the crystalline nature of the samples. The XRD pattern of CSCaP samples showed no crystalline signals, while that of CSCaPC showed intense bands at 2θ of around 26° and 33°, indicating the preferred formation of Hap during calcination. It is worth noting that this pattern is typically found not only in stoichiometric Hap but it also appears in calcium-deficient apatites [27]. The XRD pattern indicated that a poorly crystalline Hap (*Xc* lower than 86%) with broad diffracted peaks was produced after monolith calcination at 650 °C. 

After examining the XRD pattern, the question arose as to what calcium phosphate type, e.g., poorly crystalline or ACP, was formed in the CSCaP monoliths. The ^31^P MAS NMR spectra of CSCaP samples exhibited a signal at around 2.8 ppm. This signal is typically attributed to PO_4_^3^- groups from amorphous calcium phosphate (ACP) or low crystalline Hap [26]. The results of ^1^H→^31^P cross-polarization (CP) experiments revealed important differences between calcined and non-calcined samples (Figure 5). Non-calcined samples exhibited fast polarization rates, while calcined ones showed lower rates. A similar pattern has been previously described in the case of enamel and dentin, and can be explained by differences in the microstructure of the samples. Calcined samples showed a pattern similar to enamel (highly crystalline apatite mineral, ca. 96 wt %), while non-calcined samples showed a pattern similar to dentin (partially amorphous apatite, which also contains an organic matrix and water) [28]. Previous ^31^P CP MAS studies on ACP composites have reported the full suppression of the signal at large contact times [29]. This was not observed in the current case, so the presence of poorly crystalline calcium phosphate could not be fully disregarded. Nonetheless, given the morphological resemblance observed in the TEM micrographs between our non-calcined samples and those morphologies typically assigned to ACP, e.g., cluster-like structures [29,30], we can conclude that ACP was the most plausible form of CaP entrapped within the CS matrix in CSCaP monoliths. The presence of ACP was an excellent result for the application of CSCaP composites for bone repair. It is worth noting that scaffold materials used in tissue engineering need to be non-toxic and possess good biocompatibility, so we next tested whether CSCaP composites exhibited these features. The amount of calcium phosphate in the CSCaP composites strongly affected the efficacy of cell proliferation (Figure 6); i.e., the larger the calcium phosphate content of the composite, the better the cell attachment and proliferation. These results were in agreement with previous studies. It is well known that premyoblastic and preosteoblastic cells attachment and proliferation depend on the chemical composition of the sample’s surface [4]. In this case, the CaP salts acted as suitable precursors for bone tissue regeneration, and the CaP-enriched scaffolds were capable of up-regulating the first adhesion steps [3]. Moreover, it is well known that both materials (CS and CaP salts) permit the development of controlled delivery devices (including growth factors or antibiotics among other molecules of interest) [31,32,33,34]. In fact, previous studies using biocomposites containing nano-sized Hap had a great effect on the early stages of osteoblast behaviour (cell attachment and proliferation) [35]. In our case, the addition of ACP, i.e., nano-Hap, into a CS scaffold also played a similar role; it improved cell attachment, proliferation, and cell morphology when compared to the CS scaffold alone [36]. Currently, one of the main objectives in the design of biomaterials is to reproduce the physical and chemical characteristics of the damaged tissue. Cortical bone is a highly compact matrix, mainly based in ordered and cylindrical osteons, which gives support and strength. In contrast, cancellous bone is presented as an interconnected network of trabeculae where bone marrow is localized. In our composites, the structure, with porosities of around 80–85%, mimicked the native architecture of bone trabecular tissue, whereas certain resemblances between chitosan and ECM glycosaminoglycans (GAGs) contribute to the grafting capabilities of the composites being able to approach those of original bone [37]. With regard to the porous structure, it is worth noting that angiogenesis and bone ingrowth—both prerequisites to enhance tissue regeneration—will be also promoted [38]. Bone trabecular repair was actually observed in a previous work using monoliths with a similar architecture and containing bone morphogenetic protein (BMP-2) [39].

## 4. Materials and Methods 

### 4.1. Materials

Low molecular weight chitosan (CS, batch number 06513 AE, viscosity 185 cps and deacetylation degree of 90.8% as described by the supplier) and hydroxyapatite (Hap) were supplied by Aldrich (St. Luis, MO, USA). Other reagents were of analytical grade.

### 4.2. Preparation of CSCaP Composites

A CS solution (2.5% *w*/*v*) in acetic acid 0.2 M (pH 4.5) was mixed with different volumes of Hap solution (70 mg/mL) in HCl 1M (pH 3.0) under vigorous stirring in an ice-cold bath. The mixture was loaded into insulin syringes and unidirectionally frozen at 77 K in a liquid nitrogen bath at a rate of 5.9 mm/min. The freezing rate and freezing direction was controlled using homemade dip-coating equipment. The frozen samples were cut in cylinders of 10 mm and freeze-dried (ThermoSavant Micro Modulyo^®^ freeze-drier, Madison, WI, USA). Samples with different CS/Hap ratios denoted as CSCaP1, CSCaP2 and CSCaP3 were produced (Table 1).

Samples were calcined at 650 °C for 4 h in air at a heating rate of 1 °C/min and cooled at 10 °C/min (samples denoted as CSCaPC). In order to better study the nature of the mineral phase in the CSCaP composites, samples were treated in a commercial oxygen-plasma etching stripper (Tetra Pico, Diener Electronics, Ebhausen, Germany) for the elimination of organic matter in mild conditions of temperature (e.g., 20 °C) for 6 h, until no weight loss was observed (samples denoted as CSCaPOP). The chamber was first evacuated to an ultimate pressure of about 0.12 mbar. Then, commercially available oxygen was leaked into the discharge chamber. The pressure was fixed at 0.4 mbar during the experiment. The plasma generator worked at a frequency of 40 kHz. The power used in the experiments was about 300 W. With this treatment, the polymer was partially removed without altering the nature of the mineral phase.

### 4.3. Sample Characterization

The morphology of the samples was investigated by scanning electron microscopy (SEM, Carl Zeiss, Oberkochen, Germany) with a Zeiss DSM-950 instrument and by transmission electron microscopy (TEM, Jeol, Peabody, MA, USA) with a 200-KeV JEOL 2000 FXII instrument. For SEM studies, samples were mounted on a stub of metal with adhesive and coated with gold. For TEM studies, samples were milled and dispersed in acetone with sonication. One drop of the solution was put in the grid and the solvent was evaporated prior to sample examination. 

Fourier transform infrared spectra (FTIR) were collected by a NICOLET 20 SXC FTIR (ThermoFisher, Madison, WI, USA). Samples were milled previous to sample analysis. Mercury porosimetry measurements were carried out using a Micromeritics Autopore II 9220 mercury porosimeter (Micromeritics Instrument Corp., Norcross, GA, USA) following the standard procedure.

Thermogravimetric analysis (TG/DTA) was carried out with a SEIKO TG/ ATD 320 U SSC 5200 (SEIKO instruments, Chiba, Japan). Samples were heated from 20 °C to 650 °C at a heating rate of 5 °C/min under air flow (20 mL/min). XRD patterns were obtained with a Bruker D8 advance diffractometer (Bruker, Billerica, MA, USA) using Cu Kα radiation (step size 0.05°, counting time 3.5 s).

The peak broadening of XRD reflection was used to estimate the crystallite size (Xs) in a direction perpendicular to the crystallographic plane using Scherrer’s formula (Equation (1)):

(1) $$Xs=\frac{0.9\lambda }{FWHM.cos\theta }$$

where *Xs* is the crystallite size (nm), λ is the wavelength of the X-ray beam (λ = 0.15406 nm for Cu Kα radiation), FWHM is the full width at half maximum of the diffraction peak under consideration (rad), and θ is the diffraction angle. The diffraction angle around 2θ = 26° (peak assigned to (002) Miller´s plane family) was selected for the calculation of the crystallite size, since it has large intensity and is isolated from the others.

The fraction of the crystalline phase (*Xc*) in the Hap was evaluated by the following equation (Equation (2)) [32]:

(2) $$Xc=1-\frac{V112/300}{I300}$$

where *I*300 is the intensity of (300) diffraction peak and *V*112/300 is the intensity at the valley between (112) and (300) diffraction peaks of Hap. 

The splitting factor (SF), as an indication of crystallinity, was calculated from the FTIR measurements using the method given by Weiner and Bar-Yosef [22]. After a baseline correction between 400 and 800 cm^−1^, the intensity of the two PO_4_^3−^ vibration bands at 603 cm^−1^ (a) and 571 cm^−1^ (b) in the absorbance mode was measured and their sum was then divided by the intensity (c) of the valley between these absorption bands above the baseline; i.e., SF = (a + b)/c. The software Origin 7 was used for calculations.

^31^P NMR spectra of calcined and non-calcined samples were acquired using a Bruker AVANCE 200 spectrometer (Bruker, Billerica, MA, USA) with a 4-mm Bruker probe operating at 9.4 T and 162 MHz. The amount of sample used was around 5 mg. The spinning frequency was 10 kHz for the magic angle sample spinning (MAS) RMN and 7 KHz for cross-polarized MAS (CP-MAS) NMR. The contact time for ^31^CP-MAS NME ranged from 0.1 to 10 ms. The 90° pulse length was 6 µs, the repetition time was 5 s and the number of scans was 800.

### 4.4. In Vitro Cell Culture and Seeding

Cell culture C2C12-GFP (ATCC^®^ CRL-1772™, Manassas, VA, USA) mouse premyoblastic cells were treated with a lentivirus to integrate a GFP gene into the C2C12 genome. Cells were cultured in high glucose Dulbecco’s Modified Eagle Medium (DMEM), supplemented with 10% phosphate saline buffer (FBS), (Hyclone™, Fisher Scientific, Waltham, MA, USA) plus antibiotics (100 U/mL penicillin and 100 μg/mL streptomycin sulphate, Sigma-Aldrich, St. Louis, MO, USA). MC3T3 cell line (ATCC^®^ CRL-2593™, Manassas, VA, USA) is a mouse preosteoblastic cell line. Routine passaging of the cell line was performed in 25-cm^3^ flasks and cells were maintained in complete α-MEM (A10490, Gibco, UK) without ascorbic acid supplemented with 10% fetal bovine serum, (Hyclone™, Fisher Scientific, Waltham, MA, USA) plus antibiotics (100 U/mL penicillin and 100 μg/mL streptomycin sulphate, (Sigma-Aldrich, St. Louis, MO, USA). For both cell lines, culture conditions were 37 °C in a humidified 5% CO_2_ atmosphere. Cell passaging was performed when cell growth reached ≈ 80% confluence. 

CSCaP samples were cut to form cylinders of similar size and were placed into the wells of a standard 24-well plate. Samples were washed three times with phosphate buffer solution, after which the hydrogels were exposed to UV irradiation for 40 min for sterilization. After sterilization, the samples were washed three times with culture medium (DMEM) prior to cell seeding. Cells were seeded at a density of 10,000 cells/sample and a minimum of three monoliths of each sample type was used for each experiment. Afterwards, 1 mL of pre-warmed complete culture medium was added to each well and all the samples were housed in a humidified 37 °C, 5% CO_2_ atmosphere incubator. Samples were observed and fluorescent images were captured at specific time points using an inverted fluorescence microscope (Olympus IX51, Olympus, Melville, NY, USA).

### 4.5. Cell Morphology

Cells on the material were fixed with 4% paraformaldehyde (PFA) solution for 15 min. After PFA was removed, cells were rinsed with phosphate saline buffer twice and permeabilized with 0.1% (*v*/*v*) Triton X-100, washed with PBS again and stained with Texas Red^®^-X phalloidin (ThermoFisher, Madison, WI, USA), a high-affinity F-actin probe conjugated to red fluorochrome, for 20 min at room temperature in darkness. Finally, fluorescent-labelled cells were observed using an inverted fluorescence microscope (Olympus IX51, Olympus, Melville, NY, USA) with a TRICT filter (λex/λem = 550/600 nm) for Actin using CellD analysis software (Olympus, Olympus, Melville, NY, USA).

### 4.6. Cell Proliferation

Metabolic activity of cell cultures was measured by Alamar Blue assay following the manufacturer’s instructions (Biosource, Camarillo, CA, USA). This method is non-toxic, scalable and uses the natural reducing power of living cells, generating a quantitative measure of cell viability and cytotoxicity. Briefly, Alamar Blue dye (10% of the culture volume) was added to each well, containing living cells seeded over films, and incubated for 90 min. Assays were performed on each sample type in triplicate. The fluorescence (λex/λem 535/590 nm) of each well was measured using a platereader (Synergy HT, Biotek, Winooski, VT, USA).

## 5. Conclusions

In this paper, we prepared a chitosan-based macroporous composite containing amorphous calcium phosphate (ACP) salts by, first, dissolving commercial Hap in a chitosan solution and then applying the ISISA process. The formation of ACP was particularly interesting because it exhibits better in vivo osteoconductivity and biodegradability than other mineral phases, e.g., tricalcium phosphate and hydroxyapatite. Moreover, ACP can increase alkaline phosphatase activities of mesoblasts, thus enhancing cell proliferation and promoting cell adhesion. Finally, it is also worth noting that the composite’s structure, with porosities of around 80-85%, mimicked the native architecture of bone trabecular tissue, whereas certain resemblances between chitosan and ECM glycosaminoglycans (GAGs) contributed to the grafting capabilities of the composite being able to approach those of original bone. Under these premises, our composite became a quite promising candidate for tissue repair and regeneration. In fact, the studies carried out in this work with premyoblastic and preosteoblastic cell lines demonstrated how the ACP content in the composite was a key parameter for cell proliferation; i.e., increasing the ACP content from 6 to 32 wt % resulted in a remarkable increase in the metabolic activity of premioblastic cells (five-fold) and preosteoblastic cells (three-fold). Based on these preliminary in vitro results, an in vivo animal model response will be evaluated in future studies to assess the potential clinical application of this composite. 

## Figures and Tables

**Table 1:** Sample preparation conditions of CSCaP composites and thermogravimetric analysis.

Sample	CS/CaP Weight Ratio	Theoretical Mineral Content, %	Humidity at 55 °C, % ^1^	Residual Weight at 650 °C, % ^1^
CSCaP1	56:3.75	6.25	8.9	7.3
CSCaP2	56:14	20.0	9.0	19.4
CSCaP3	56:26.3	31.9	8.0	20.3

^1^ As determined by thermogravimetric analysis of the samples.

**Table 2:** Effect of CaP content on the crystallinity and crystallite size of CaP in the CSCaPC monoliths.

Sample	Crystallinity, *Xc* (%) ^1^	Splitting FactorSF ^2^	Crystallite Size, Xs (nm) ^1^
CSCaP1C	76	3.5	18
CSCaP2C	86	4.1	21
CSCaP3C	--	3.2	17

^1^ Determined from XRD analysis. ^2^ Determined from FTIR analysis.

## Supplementary Materials

The following are available online at www.mdpi.com/1996-1944/10/5/516/s1. Figure S1: Thermogravimetric analysis of CSCaP composites. (A) CSCaP1; (B) CSCaP2 and (C) CSCaP3 samples. Figure S2: XRD pattern of (A) Chitosan; (B) Hydroxyapatite and (C) physical mixture of chitosan and hydroxyapatite. Figure S3: FTIR spectra of CSCaP1OP (A) and CSCaP1C (B) samples. Figure S4: Typical ^31^P NMR spectra of CSCaP (A) and CSCaPC (B) samples. Figure S5: Fluorescence microscopy of premyoblastic C2C12-GFP cell culture at 7days. (A) CS scaffold; (B) CSCaP1 composite; (C) CSCaP2 composite and (D) CSCaP3 composite. Figure S6: Actin staining of osteoblastic MC3T3 cell culture at 7 days. (A) CS scaffold; (B) CSCaP1 composite; (C) CSCaP2 composite and (D) CSCaP3 composite.
