# Supplementary material for: Macroporous Calcium Phosphate/Chitosan Composites Prepared via Unidirectional Ice Segregation and Subsequent Freeze-Drying
Source: Materials (Basel). 2017 May 8;10(5):516. doi: 10.3390/ma10050516 (PMC5459033; doi:10.3390/ma10050516)
Supplement: Supplementary file 1 [file materials-10-00516-s001.pdf]

Supplementary Materials: Macroporous Calcium Phosphate/Chitosan Composites Prepared via Unidirectional Ice Segregation and Subsequent Freeze-Drying

Inmaculada Aranaz, Enrique Martínez-Campos, Carolina Moreno-Vicente, Ana Civantos, S. García-Arguelles and Francisco del Monte

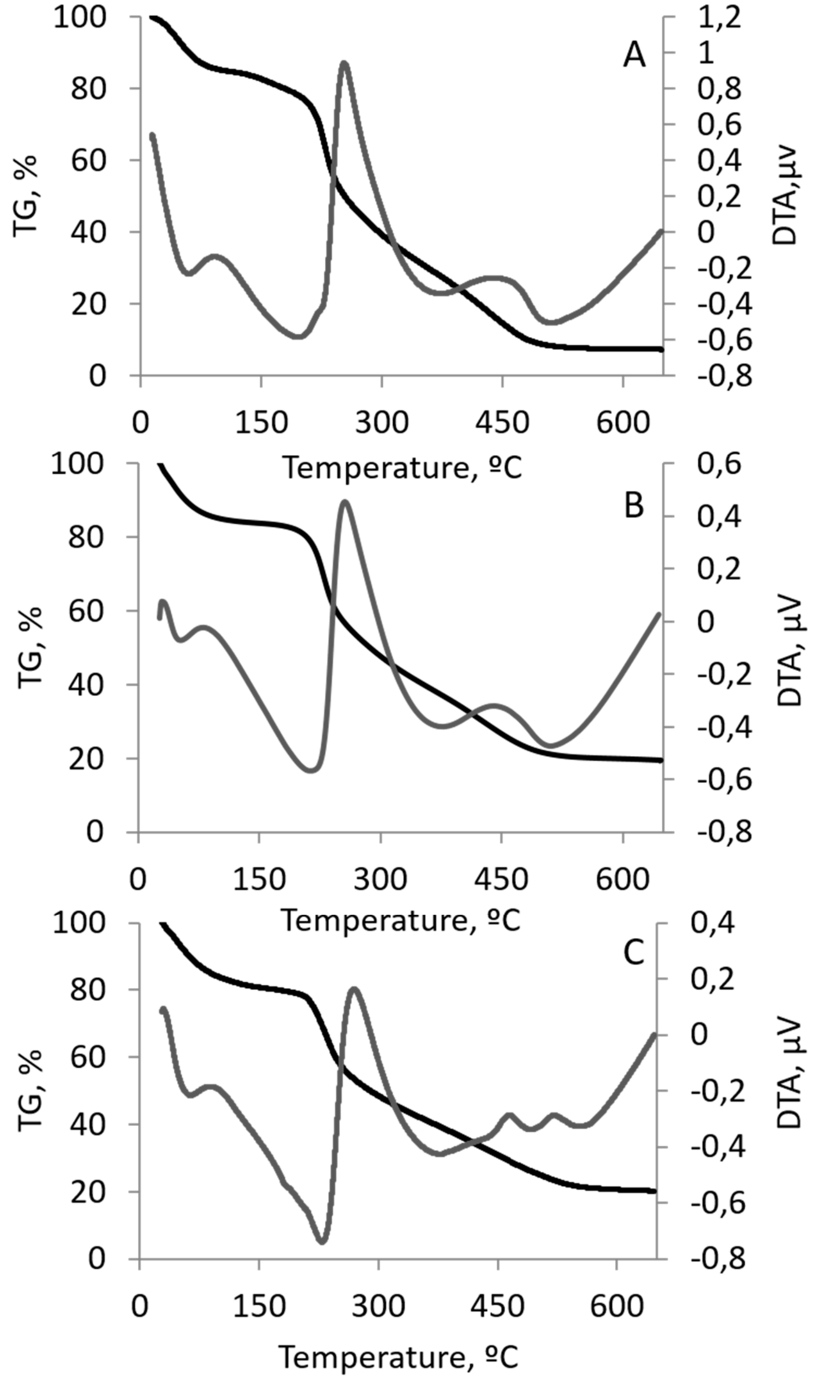

Figure S1. Thermogavimetric analysis of CSCaP composites. (**A**) CSCaP1, (**B**) CSCaP2 and (**C**) CSCaP3 samples.

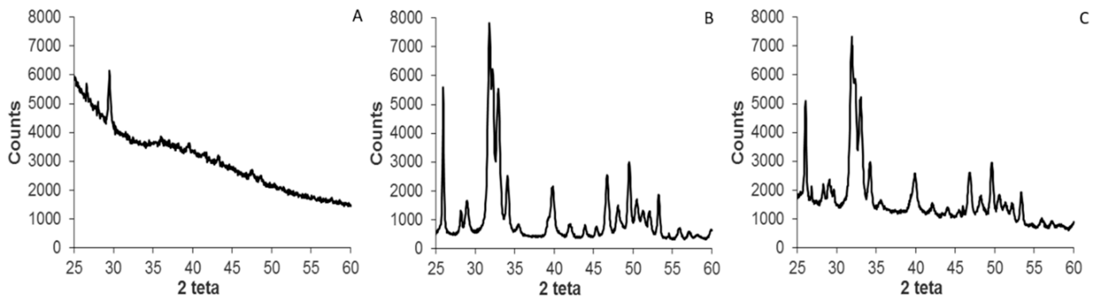

Figure S2. XRD pattern of (**A**) chitosan, (**B**) hydroxyapatite and (**C**) physical mixture of chitosan and hydroxyapatite.

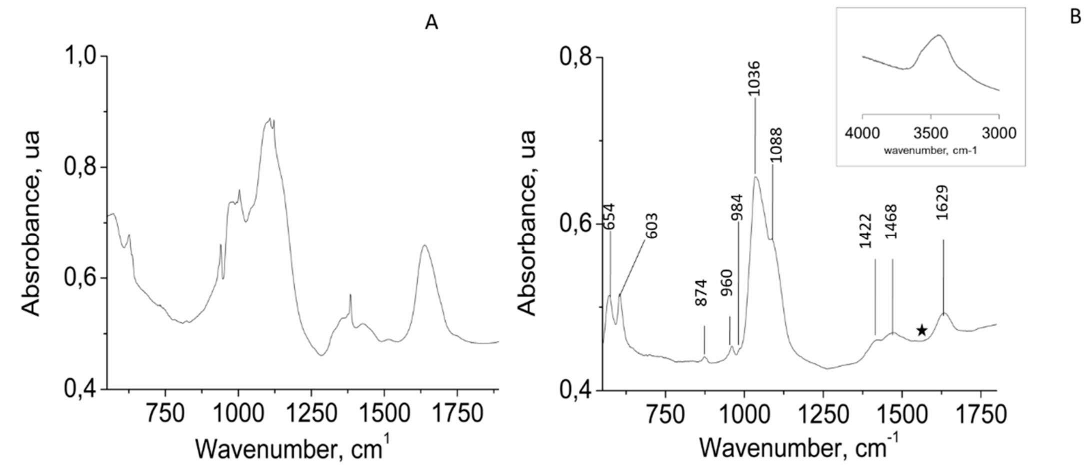

Figure S3. FTIR spectra of CSCaP1OP (**A**) and CSCaP1C (**B**) samples.

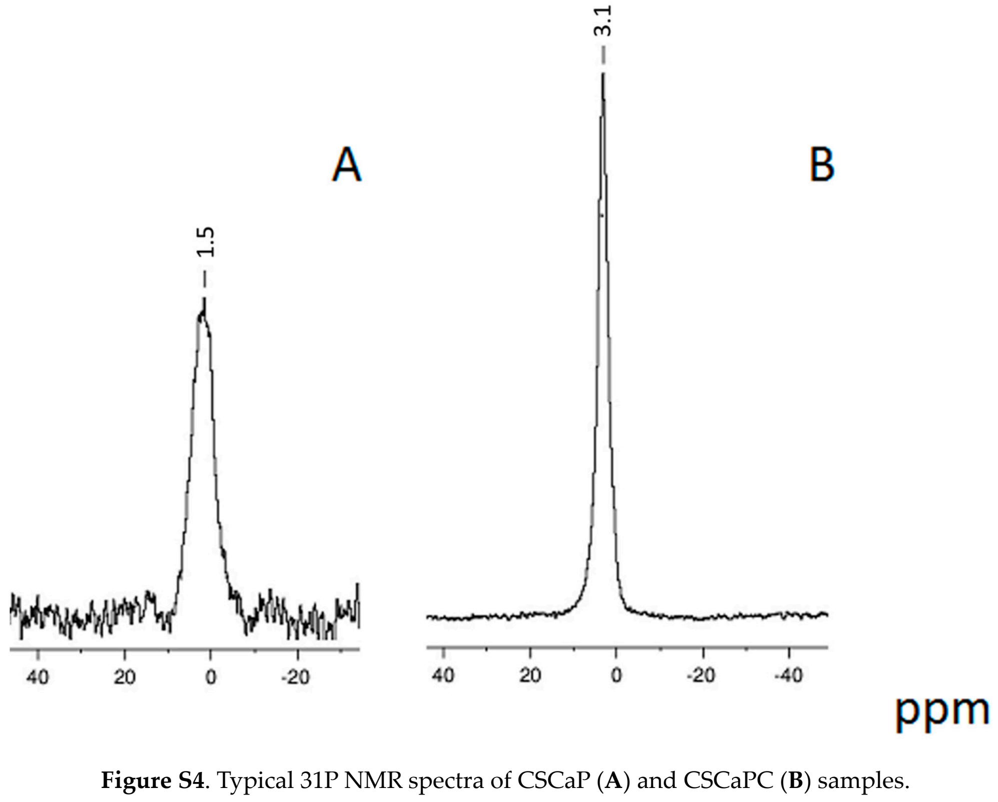

Figure S4. Typical 31P NMR spectra of CSCaP (**A**) and CSCaPC (**B**) samples.

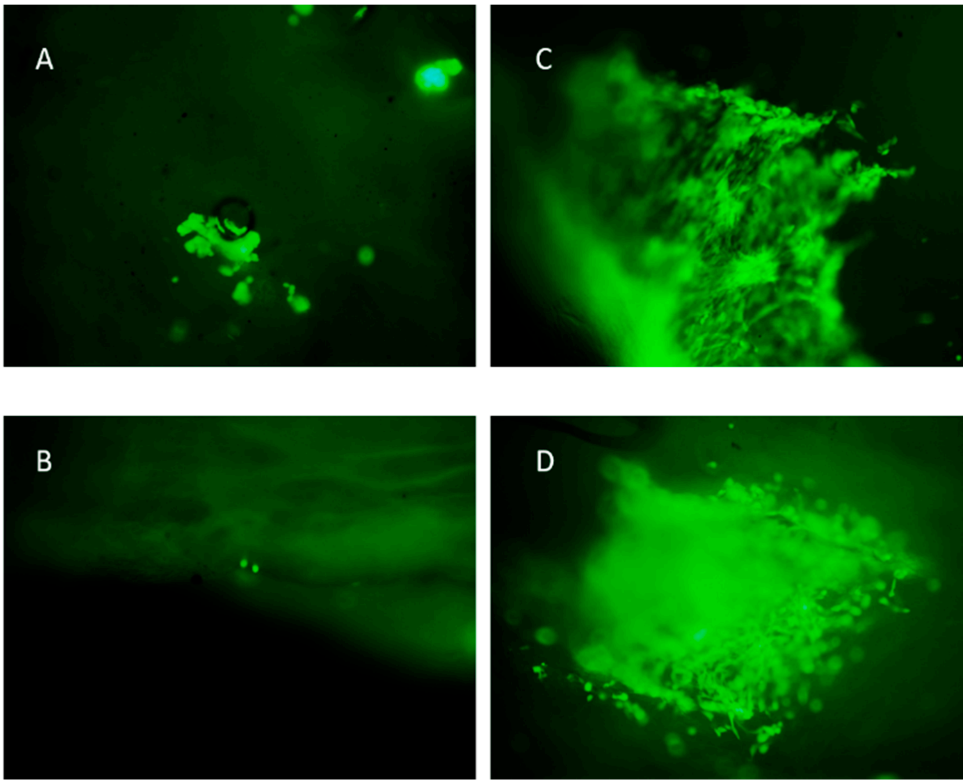

Figure S5. Fluorescence microscopy of premyoblastic C2C12-GFP cell culture at 7days. (**A**) CS scaffold, (**B**) CSCaP1 composite; (**C**) CSCaP2 composite and (**D**) CSCaP3 composite.

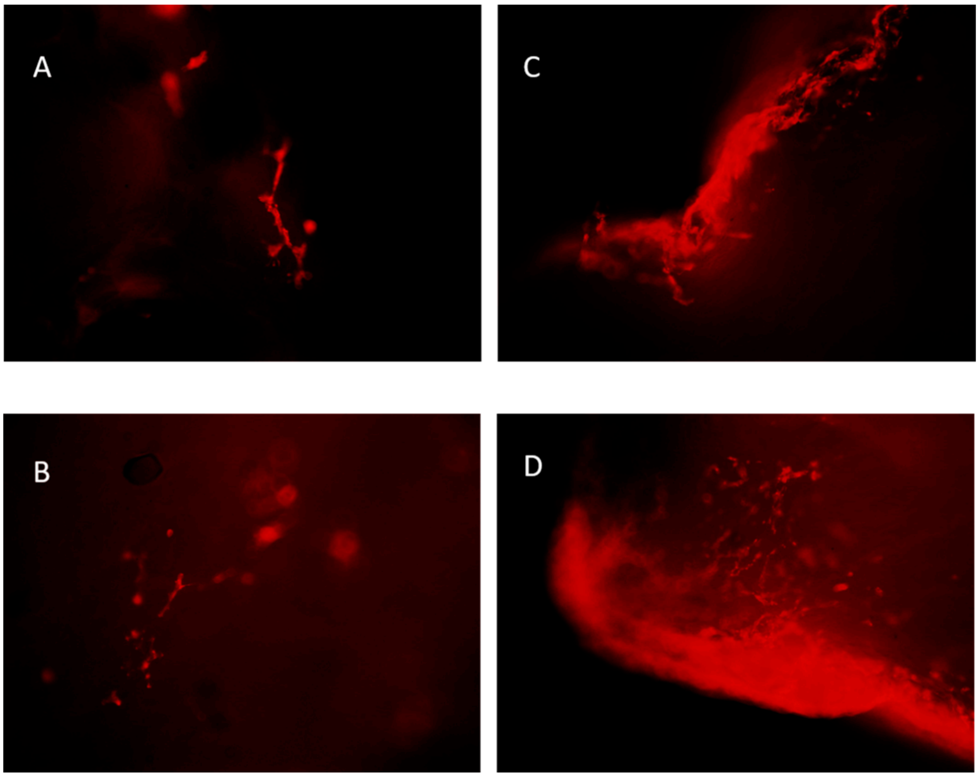

Figure S6. Actin staining of osteoblastic MC3T3 cell culture at 7 days. (**A**) CS scaffold, (**B**) CSCaP1 composite; (**C**) CSCaP2 composite and (**D**) CSCaP3 composite.
